# Awareness and attitudes amongst basic surgical trainees regarding radiation in orthopaedic trauma surgery

**DOI:** 10.2349/biij.6.3.e25

**Published:** 2010-07-01

**Authors:** FR Khan, Z Ul-Abadin, S Rauf, A Javed

**Affiliations:** 1 Ysbyty Gwynedd, Penrhosgarnedd, Bangor, Gwynedd, United Kingdom; 2 Blackpool Victoria Hospital, Blackpool, Lancashire, United Kingdom

**Keywords:** radiation protection, surgical trainees, training

## Abstract

This study investigated the awareness and attitudes of basic surgical trainees. Trainees were asked to answer questions from a pre-set questionnaire. Fifty basic surgical trainees from England and Wales were involved in the study. The areas covered were basic knowledge of radiation hazards, use of protective wear, pregnancy test in female trauma victims of reproductive age, and principles of safe radiation. All the questions were asked in the context of orthopaedic trauma surgery. All questions were evidence based.

It was unfortunate to notice that basic surgical trainees are lacking in the essential knowledge of ionising radiation. Most of the trainees are not adhering to radiation safety principle, and are not practising safely. The authors strongly recommend that surgical trainees should have more robust training and information available in this context. And they suggest that it should be provided on local, regional and national basis. © 2010 Biomedical Imaging and Intervention Journal. All rights reserved.

## INTRODUCTION

Modern orthopaedics is becoming increasingly characterised by operative intervention, especially trauma surgery, where x-rays (fluoroscopy) are used. With the ever-growing knowledge and awareness of radiation amongst patients, it is crucial that surgical trainees get more awareness of ionising radiation. A literature search revealed many studies regarding exposure of orthopaedic surgeons (both junior and senior) to radiation. But no study has been done in the context of the trainees’ awareness of ionising radiation. The authors therefore decided to assess the awareness and attitudes of the basic surgical trainees towards ionising radiation in orthopaedic trauma surgery.

## MATERIALS AND METHODS

**Figure 1:**
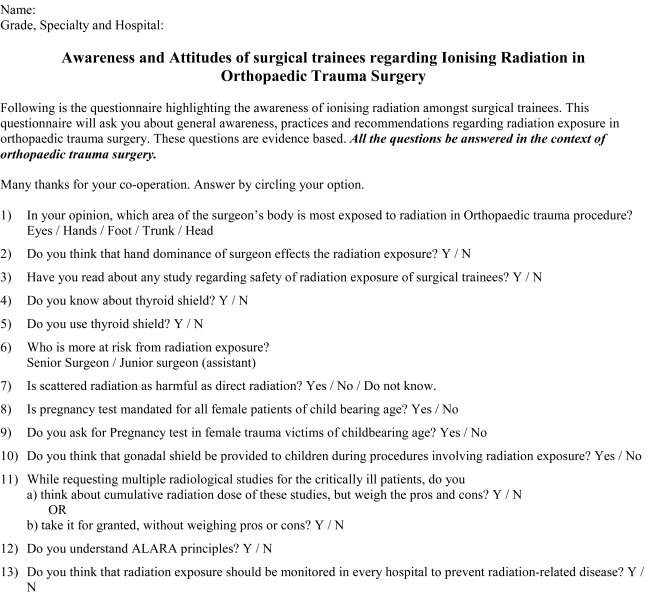
Questionnaire on ionising radiation for surgical trainees.

Fifty basic surgical trainees were involved in the study, and were asked to complete a questionnaire (Figure 1). All the questions were evidence based [1-14], and covered the following topics:

- knowledge of the area of the body most exposed to radiation in orthopaedic trauma surgery
- hand dominance and its effects on radiation exposure
- knowledge of any literature of about radiation safety
- knowledge of the thyroid shield and its usage
- grade of surgeon and risk of radiation exposure
- pregnancy test in female patients of childbearing age
- gonadal shield in children
- multiple radiological studies for critically ill patients and their impact on deciding the number of radiological studies (cumulative radiation dose effect)
- knowledge of ALARA principle
- monitoring of radiation exposure

## RESULTS

Fifty basic surgical trainees were involved in the study. Their responses to the 13 questions are tabulated in Table 1.

**Table 1:** Presentation of results

Questions	Results
Most affected area for exposure	Hand: 25; Head: 16; Trunk: 6; Eyes: 3
Scattered versus Diffuse radiation severity	Scattered: 15; Diffuse: 11; No knowledge: 24
Radiation exposure to senior or junior	Seniors: 15; Juniors: 30; No difference: 5
Effect of hand dominance exposure	Yes: 23; No: 25
Literature reading for radiation safety	Yes: 8; No: 42
Awareness of thyroid shield	Yes: 44; No: 6
Use of thyroid shield	Yes: 12; No: 38
Pregnancy test in trauma patients	Yes: 34; No: 16
Use of gonadal sheet	Yes: 47; No: 3
Pros and cons of x-ray radiation	Yes: 30; No: 20
Awareness of ALARA principle	Yes: 3; No: 47

## DISCUSSION

There are plenty of studies done on radiation exposure in orthopaedic trauma surgery [1-14], but only eight out of 50 surgical trainees from this study population had read any literature about it. Smith et al. reported that hand exposure to radiation is a limiting factor in orthopaedic trauma surgery. This differs from the previously studied groups, such as cardiologists and radiologists, in whom the limiting factor is the dose to the lens of the eye [1]. Jones et al. advised the orthopaedic surgeons to use dose reduction gloves for high risk procedures [2].

Sanders et al. found in their study that there is no positive correlation between the hand dominance of the surgeon and radiation exposure to hands [2]. They used gas sterilised thermoluminescent dosimeter ring worn on each hand. The rings were later submitted for dose evaluation. In this study, 23 trainees thought that hand dominance does affect radiation exposure.

Bahari et al. and Muller et al. discussed the significance of the thyroid shield in orthopaedic trauma surgery. Bahari showed that there was significant difference in the unshielded thyroid groups as compared to the shielded thyroid group (p<0.05) [4]. Muller and colleagues discussed the effectiveness of lead thyroid shield in reducing x- ray exposure in trauma surgery interventions of the lower leg. They concluded that the average registered ionising dose without thyroid shield was 70 times higher as compared to the measurement with thyroid protection [5]. Alonso concluded that the thyroid shield should be made available to operating staff within a 2-metre zone [6]. Herscovici also advised surgeons to wear protective devices [7].

Tasbas et al. reported that the assistant surgeon is more at risk than the senior surgeon [8]. In the study, they found that the orthopaedic surgeon was always standing at a safe distance (>90cm), but the assistant surgeon always stood nearby (10 cm) to the x-ray source for positioning of the patient. The reading on the badges of the assistant surgeon was more than the orthopaedic surgeon. In this study, 15 trainees thought that senior surgeons received higher level of radiation exposure. Thirty trainees thought otherwise, while five trainees thought it to be equal exposure.

Alonso et al. studied the effects of scattered radiation during hip fracture fixation and considered that beyond 2 metres from the radiation source, the scattered dose received was consistently low while within the operating distance, the scattered dose received by staff was high for both lateral and anteroposterior projections [6]. Herscovici advised surgeons to increase their distance from the x-ray beam to reduce the risk from radiation [7]. In this study, 24 trainees did not know the difference between scattered and direct radiation while 15 trainees considered scattered radiation to be as harmful as direct radiation. Only 11 trainees did not consider scattered radiation to be as harmful as direct radiation.

Flik et al. recommended that pregnancy test is mandatory for all females of childbearing age who are involved in trauma. They reported that trauma affects up to 8% of pregnancies, and is the leading cause of death among pregnant women in the United States [9]. Bochicchio et al. concluded that rapid pregnancy test should be done in all female trauma victims of childbearing age. They were of the view that trauma patients diagnosed with incidental pregnancy (pregnancy status unknown to the trauma team) are routinely exposed to doses of radiation exceeding the recommendations of the American College of Obstetrics and Gynecologists [10]. In their study of 3,976 women of reproductive age admitted in trauma centre, 13 (11.4%) were found to be pregnant incidentally. Foetal mortality in these patients was significantly higher than others (10 out of 13, 77%) [10]. Mann and colleagues argued that trauma surgeons must balance the risks and benefits of diagnostic radiographic procedures on potentially pregnant patients, and should know the range and likelihood of possible radiation effects on pregnancy [11]. In this study, 34 trainees considered it mandatory while 16 did not. When asked whether a pregnancy test will be requested in such cases, 38 responded positively.

Gul et al. found out that children receive many radiographs with avoidable excess radiation from inadequate positioning or complete omission of gonadal shields. In their opinion, this may increase the potential for disease in the future offspring of these patients. They concluded that strict adherence to guidelines is required to decrease the radiation exposure in children [12]. Their view was supported by Herscovivi [7].

In a study conducted in an urban level 1 trauma centre in the United States, 46 trauma patients who stayed for more than 30 days in a surgical intensive care unit were studied for cumulative effective dose (CED) of radiation from radiologic studies. The mean CED in this study group was 30 times higher than the average yearly radiation dose from all U.S. sources [13]. In this study, 30 surgical trainees said that they weighed the pros and cons of radiation while requesting multiple radiological studies in critically ill patients while the remaining 20 trainees were of the opinion that they take it for granted, and request the x-rays without weighing pros and cons.

Bahari recommended that orthopaedic surgeons should adhere to ALARA principles [4]. ALARA stands for As Low As Reasonably Achievable. It means that while requesting x-rays (diagnostic, as well as fluoroscopy in theatres), surgeons should request as less as reasonably possible. Herscovici advised the orthopaedic surgeons to limit the radiation exposure [7]. Oddy concluded that the principle of minimising radiation exposure must be maintained by all trainees at all times [14]. Unfortunately, most (47 out of 50) trainees in this study were unaware of the ALARA principles.

Bahari also recommended that routine monitoring of radiation exposure is essential in preventing radiation-related diseases [4]. Sanders argued that extremity dosimetry for surgeons regularly using x-ray should be considered [3] while Herscovici advised that radiographic units should undergo periodic calibration [7]. In this study, 46 trainees liked the idea of routine monitoring of radiation.

## CONCLUSION

The results of this study show that the basic surgical trainees are lacking in the essential knowledge of ionising radiation in orthopaedic trauma surgery. Most of them had never read the literature about it. Most of the surgical trainees did not wear the thyroid shield, and some of them were even unaware of it. Most of the trainees did not know the difference between scattered and direct radiation. One-third of trainees did not consider pregnancy test to be mandatory. Even worse, one-fourth of trainees did not even ask for a pregnancy test. Two-fifths of the trainees requested radiological studies without weighing the pros and cons. The majority (47 out of 50) did not know about the safety principle for radiation.

Based on the above facts, the authors recommend that basic surgical trainees should have more information and knowledge about the ionising radiation. The courses should be arranged at local as well regional and national level. They trust this course can be included in their induction for junior doctors. This will not only help the professional competence of surgical trainees, but it will make them safe doctors as well.

## References

[1] Smith GL, Briggs TW, Lavy CB (1992). Ionising radiation: are orthopaedic surgeons at risk?. Ann R Coll Surg Engl..

[2] Sanders R, Koval KJ, DiPasquale T (1993). Exposure of the orthopaedic surgeon to radiation. J Bone Joint Surg Am..

[3] Jones DG, Stoddart J (1998). Radiation use in the orthopaedic theatre: a prospective audit. Aust N Z J Surg..

[4] Bahari S, Morris S, Broe D (2006). Radiation exposure of the hands and thyroid gland during percutaneous wiring of wrist and hand procedures. Acta Orthop Belg..

[5] Muller LP, Suffner J, Mohr W (1997). [Effectiveness of lead thyroid shield for reducing roentgen ray exposure in trauma surgery interventions of the lower leg]. Unfallchirurgie..

[6] Alonso JA, Shaw DL, Maxwell A (2001). Scattered radiation during fixation of hip fractures. Is distance alone enough protection?. J Bone Joint Surg Br..

[7] Herscovici D, Sanders RW (2000). The effects, risks, and guidelines for radiation use in orthopaedic surgery. Clin Orthop Relat Res..

[8] Tasbas BA, Yagmurlu MF, Bayrakci K (2003). Which one is at risk in intraoperative fluoroscopy? Assistant surgeon or orthopaedic surgeon?. Arch Orthop Trauma Surg..

[9] Flik K, Kloen P, Toro JB (2006). Orthopaedic trauma in the pregnant patient. J Am Acad Orthop Surg..

[10] Bochicchio GV, Napolitano LM, Haan J (2001). Incidental pregnancy in trauma patients. J Am Coll Surg..

[11] Mann FA, Nathens A, Langer SG (2000). Communicating with the family: the risks of medical radiation to conceptuses in victims of major blunt-force torso trauma. J Trauma..

[12] Gul A, Zafar M, Maffulli N (2005). Gonadal shields in pelvic radiographs in paediatric patients. [Bulletin].

[13] Kim PK, Gracias VH, Maidment AD (2004). Cumulative radiation dose caused by radiologic studies in critically ill trauma patients. J Trauma..

[14] Oddy MJ, Aldam CH (2006). Ionising radiation exposure to orthopaedic trainees: the effect of sub-specialty training. Ann R Coll Surg Engl..

